# Adverse Newborn Outcomes by Insurance Status Among Patients with Severe Maternal Morbidity in Maryland: 2020–2023

**DOI:** 10.3390/healthcare14060804

**Published:** 2026-03-21

**Authors:** Porcia Manandhar, Carrie Wolfson, Jeanne Sheffield, Michelle Phillips, Ernest Graham, Robert Atlas, Pamela Chin, Joanne Olaku, Robyn Duafala, Brittany L. Cline, Irina Burd, Jenifer Fahey, Kimberly Jones-Beatty, Krista M. Mehlhaff, Monica B. Jones, Kathryn Buchanan, Megan E. Carey, Jan Chiang, Cynthia Argani, Eva Kelly, Kelly Krout, Ichchha Madan, Cathy Downey, Jennifer Kasirsky, Amber M. Richter, Hannah Starr, James L. Wynn, Andreea A. Creanga, Khyzer B. Aziz

**Affiliations:** 1 Department of International Health, Johns Hopkins Bloomberg School of Public Health, Baltimore, MD 21205, USA; cwolfson@som.umaryland.edu (C.W.); acreanga@som.umaryland.edu (A.A.C.); 2 Department of Gynecology and Obstetrics, Johns Hopkins University School of Medicine, Baltimore, MD 21205, USA; jsheffi2@jhmi.edu (J.S.); mphill44@jhmi.edu (M.P.); egraham5@jhmi.edu (E.G.); 3 Mercy Medical Center, Baltimore, MD 21202, USA; ratlas@mdmercy.com (R.A.); pchin@mdmercy.com (P.C.); 4 Sinai Hospital of Baltimore, Baltimore, MD 21215, USA; jolaku@lifebridgehealth.org (J.O.); rduafala@lifebridgehealth.org (R.D.); bcline@lifebridgehealth.org (B.L.C.); 5 Department of Obstetrics, Gynecology and Reproductive Sciences, University of Maryland School of Medicine, Baltimore, MD 21201, USA; iburd@som.umaryland.edu (I.B.); jfahey@som.umaryland.edu (J.F.); kjonesbeatty@som.umaryland.edu (K.J.-B.); kmehlhaff@som.umaryland.edu (K.M.M.); 6 Luminis Health Anne Arundel Medical Center, Annapolis, MD 21401, USA; mjones23@luminishealth.org (M.B.J.); kbuchanan@luminishealth.org (K.B.); mcarey@luminishealth.org (M.E.C.); jchiang@luminishealth.org (J.C.); 7 Johns Hopkins Bayview Medical Center, Baltimore, MD 21224, USA; cholcroft@jhmi.edu (C.A.); ekelly26@jhmi.edu (E.K.); kbishop4@jh.edu (K.K.); 8 Johns Hopkins Howard County General Hospital, Columbia, MD 21044, USA; imadan1@jhu.edu (I.M.); cdowney3@jhmi.edu (C.D.); 9 Adventist HealthCare Shady Grove Medical Center, Rockville, MD 20850, USA; jenniferkasirsky@gmail.com (J.K.); arichter@adventisthealthcare.com (A.M.R.); hstarr@adventisthealthcare.com (H.S.); 10 Department of Pediatrics, University of Florida, Gainesville, FL 32603, USA; 11 Department of Pediatrics, Johns Hopkins University School of Medicine, Baltimore, MD 21205, USA; kaziz5@jhmi.edu

**Keywords:** severe maternal morbidity, adverse newborn outcomes, Medicaid, comorbidities, Maryland

## Abstract

**Background:** Adverse newborn outcomes in patients with severe maternal morbidity (SMM) are understudied, and this study examines their association with insurance type (Medicaid vs. commercial) in patients who experienced SMM. The aim of this study is to examine disparities in preterm birth, low birthweight, and neonatal intensive care (NICU) admission among Medicaid vs. commercially insured patients with severe maternal morbidity in Maryland. **Methods:** This cross-sectional study analyzed data from 588 SMM patients enrolled in Maryland’s Severe Maternal Morbidity (SMM) Surveillance Program (August 2020–December 2023). We utilized unadjusted and multivariable logistic regression models to evaluate the relationship between primary insurance type and the outcomes of interest: preterm birth (<37 weeks), low birthweight (<2500 g), and neonatal intensive care unit (NICU) admissions. **Results:** Of 588 patients with SMM, 45.1% had Medicaid. These patients were younger, more often non-Hispanic Black or Hispanic, had higher parity and comorbidity scores, and initiated prenatal care later compared with commercially insured patients. Medicaid patients had 2.2 to 2.6 times higher odds of adverse newborn outcomes after adjusting for other socio-demographic and medical factors. Patients’ comorbidities significantly increased the odds of adverse newborn outcomes, as did all other primary SMM causes other than obstetric hemorrhage. **Conclusions:** Adverse newborn outcomes were more prevalent among Medicaid than commercially insured patients who experienced SMM. Differences in maternal health status and primary SMM cause partly explain the observed differences in newborn outcomes. Our findings emphasize the need for comprehensive prenatal care and improved healthcare access for women with high-risk pregnancies.

## 1. Introduction

In the United States, severe maternal morbidity (SMM) increased by 40% from 72.0 to 101.1 cases per 10,000 delivery hospitalizations between 2016 and 2021, respectively [1]. SMM is broadly defined by the Centers for Disease Control and Prevention as “*unexpected outcomes of labor and delivery that can result in significant short- or long-term health consequences*” [2]. In addition to the complications and health impacts that women with SMM have, adverse maternal outcomes can also impact outcomes for the fetus and neonate [3,4]. Such outcomes include preterm birth, small for gestational age, low Apgar score, neonatal intensive care unit (NICU) admission, and even fetal or neonatal death [5,6,7]. More specifically, a systematic review and meta-analysis conducted in high-income countries showed that infants born to women with SMM were 3.1 times more likely to be preterm, 3.7 times more likely to have a 5 min Apgar score <7, and 3.2 times more likely to require NICU admission [5].

Health insurance plays a critical role in ensuring access to prenatal and delivery care for US women. Medicaid, the public health insurance program, serves low-income individuals, covering approximately two-fifths of all US births and more than a third of births in Maryland [8]. In contrast, commercial insurance is private coverage typically provided by employers or purchased individually. Given that Medicaid has an income threshold for eligibility, women with commercial insurance coverage tend to be higher income than those with Medicaid insurance coverage. Women with Medicaid are up to 1.5 times more likely to experience SMM compared to commercially insured women [3,9]. Adverse newborn outcomes, including neonatal mortality [10,11], low birthweight [10,12,13], preterm birth [10,13,14,15], and NICU admission [10,16], also occur at higher rates among Medicaid beneficiaries than patients with commercial insurance.

Less is known about whether insurance status is associated with adverse newborn outcomes among patients with SMM. To fill this gap, we examined differences in preterm birth, low birthweight, and neonatal intensive care (NICU) admission among Medicaid vs. commercially insured patients with SMM in Maryland.

## 2. Methods

**Study Sample:** This study included pregnant and postpartum patients who experienced SMM at one of 15 birthing hospitals participating in Maryland’s SMM Surveillance and Review program between 1 August 2020 and 31 December 2023 [17]. Annual number of deliveries across these 15 hospitals during the reporting period was approximately 32,000. The sample was restricted to patients whose primary insurance provider was either commercial insurance or Medicaid during their index hospitalization with SMM. The surveillance criteria for SMM included patients with hospital admissions during pregnancy or within 42 days postpartum that involved admission to an intensive/critical care unit (ICU/CCU) and/or transfusion of 4 or more units of blood products as per the recommendations of the American College of Obstetricians and Gynecologists (ACOG) and the Society for Maternal-Perinatal Medicine (SMFM) [17,18]. Analyses of newborn outcomes were restricted to SMM events that occurred during the delivery hospitalization with deliveries resulting in live births. Because the data were de-identified public health surveillance data, the Institutional Review Board at the Johns Hopkins Bloomberg School of Public Health deemed this study as not human subjects research.

**Data Collection**: Detailed information about Maryland’s hospital-based SMM Surveillance and Review program is available elsewhere [17,18]. Briefly, a trained nurse or physician assistant identified patients meeting the SMM surveillance case definition in near real time, typically within one month of occurrence. Cases were reviewed by multidisciplinary hospital-based committees, typically consisting of obstetrician(s), quality improvement specialist(s), nursing staff, and 1–3 data abstractor(s). Review committees determined the primary cause of SMM and whether the case was potentially preventable with changes to provider, system and/or patient factors during the antepartum, intrapartum or postpartum period. Data from these patients’ electronic health records were abstracted into a standardized database, including patient socio-demographic and medical history information, primary insurance during the hospitalization with SMM, prenatal care, and delivery information and outcomes. Data abstractors also provided a case narrative and timeline related to the SMM event for all patients.

**Study Variables:** The three outcomes of interest for this analysis were preterm birth (live birth at <37 weeks’ gestation); low birthweight (birthweight < 2500 g); and NICU admission. For twin pregnancies, an outcome was determined as present if either newborn was preterm, had low birthweight, and/or required NICU admission. The main covariate of interest was primary insurance type at the index delivery hospitalization, grouped as Medicaid or commercial insurance. Other covariates included the following: maternal age at hospitalization with SMM (<25 years, 25–34 years, ≥35 years); maternal race/ethnicity (non-Hispanic Black, non-Hispanic White, Hispanic, Asian, multi-race/other/unknown); parity; newborn sex; use of assisted reproductive technology (ART) during index pregnancy (yes/no); the primary cause of SMM, including the top three causes and all other causes in aggregate (obstetric hemorrhage, hypertensive disorders of pregnancy, infection, other); SMM timing, classified as antepartum (during pregnancy before the onset of labor), intrapartum (from the onset of labor to delivery of the placenta), or postpartum (the period following delivery); and the maternal comorbidity index score adapted from Leonard et al. [19] to align with information available in the surveillance data (Supplemental Table S1). To further describe the cohort, we also explored associations between primary insurance type and timing of prenatal care initiation (1st trimester, classified as on time; 2nd or 3rd trimester, classified as late; or no prenatal care); mode of delivery (cesarean, spontaneous vaginal, assisted vaginal, and dilation and evacuation); presence of congenital anomaly (yes/no); length of hospital stay (days); and maternal ICU/CCU admission or receipt of ≥4 units of blood products during the index hospitalization.

**Figure 1:**
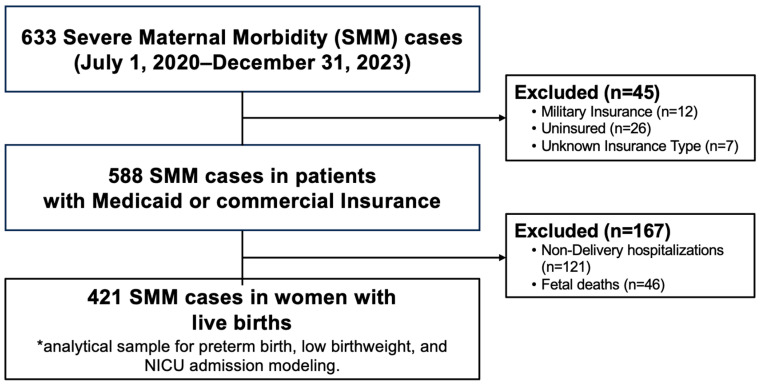
Severe maternal morbidity (SMM) event selection and analytical sample derivation (2020–2023). “*”: The group is the one used for the main analysis.

**Statistical Analysis:** We compared maternal, delivery, fetal and newborn characteristics, as well as SMM event characteristics between patients with Medicaid and commercial insurance, using chi-square analyses for categorical variables and *t*-tests for continuous variables. To assess the relationships between patients’ primary insurance type and our outcomes of interest, we fitted unadjusted and multivariable logistical regression models to an analytic sample limited to live births among patients who experienced SMM during the delivery hospitalization (Figure 1). Separate models were fitted for preterm birth, low birthweight, and NICU admission. The selection of covariates included in adjusted regression modeling was based on their associations with both insurance type and adverse newborn outcomes from the available literature [9,10,19,20]. Data were analyzed using Stata version 15 [21].

**Reporting:** This manuscript was prepared in accordance with the STROBE (Strengthening the Reporting of Observational Studies in Epidemiology) guidelines for observational studies.

## 3. Results

Across the 15 hospitals, 633 SMM events were identified and reviewed between 1 August 2020 and 31 December 2023. Of the 633 patients with SMM, 265 (45.1%) had Medicaid as their primary insurance type during their hospitalization with SMM and 323 (54.9%) had commercial insurance, for a total of 588 patients included in the analysis (Figure 1, Table 1). Among these 588 patients, 421 experienced SMM during the delivery hospitalization and had a live birth, comprising the sample used for analyses of newborn outcomes (preterm birth, low birthweight, and NICU admission).

Medicaid beneficiaries differed significantly from patients with commercial insurance—they were, on average, younger, more often non-Hispanic Black or Hispanic, had higher parity, initiated prenatal care later or not at all, and used ART less frequently, while having higher comorbidity index scores (Table 1); they also had higher rates of substance use and major mental health disorders (Supplemental Table S1).

**Figure 2:**
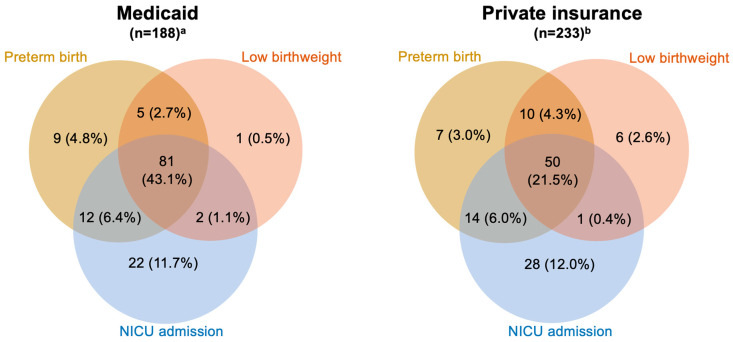
Prevalence of preterm birth, low birthweight and NICU admission among live birth deliveries in patients with SMM by insurance type in Maryland: 2020–2023. Notes: NICU, Neonatal Intensive Care Unit. ^a^ n = 56 (29.8%) had no adverse neonatal outcome; ^b^ n = 115 (49.4%) had no adverse neonatal outcome, and n = 2 (0.9%) had missing adverse neonatal outcome status.

Among the 421 live births, 44.7% were preterm, 37.6% had low birthweight, and 50.7% resulted in NICU admission (Table 1 and Table S2). Medicaid patients had significantly higher proportions of preterm birth (56.9% Medicaid versus 34.8% commercial), low birthweight (47.6% Medicaid versus 29.4% commercial), and NICU admission (63.6% Medicaid versus 40.4% commercial). Most newborns in this study had at least two of the three adverse outcomes, while 43.1% of those born to patients with Medicaid and 21.5% of those born to patients with commercial insurance experienced all three of these outcomes (Figure 2).

Patients with Medicaid and commercial insurance experienced SMM during the delivery hospitalization at similar rates (80.4% and 78.6%, respectively), yet patients with Medicaid had a longer mean length of hospital stay (7.9 days versus 6.1 days for patients with Medicaid and commercial insurance, respectively; Table 2). A significantly lower proportion of patients with Medicaid compared to commercial insurance had obstetric hemorrhage as primary SMM cause. SMM events were deemed preventable among similar proportions of patients with Medicaid and commercial insurance (33.2% and 34.4%, respectively).

Compared to patients with commercial insurance, Medicaid recipients had 2.2 to 2.6 times the odds of preterm, low birthweight and NICU admission outcomes in unadjusted regression analyses (all *p* < 0.005; Table 3). However, only the increased odds of NICU admission remained statistically significant after adjusting for maternal race/ethnicity, parity, comorbidity index, use of ART, newborn sex, primary cause and timing of SMM [Adjusted OR (AOR) = 1.83; 95% CI: 1.09–3.07] (Table 3). Relative to non-Hispanic White patients, non-Hispanic Black patients had significantly higher odds of preterm birth [OR = 1.76; 95% CI: 1.14–2.73] and low birthweight [OR = 1.72; 95% CI: 1.10–2.71] only in unadjusted analyses. Notably, across unadjusted and adjusted regression models, a higher maternal comorbidity index score was associated with significantly higher odds of neonatal adverse outcome. Specifically, for each 1-unit increase in the maternal comorbidity index score, the odds of having an adverse newborn outcome increased by 9.0% for preterm birth, 8.0% for low birthweight, and 5.0% for NICU admission in adjusted models.

Also of note, compared to patients whose primary cause of SMM was obstetric hemorrhage, after adjusting for covariates, patients with a hypertensive disorder of pregnancy as primary cause of SMM were 4.8 times more likely to deliver preterm [95% CI: 2.19–10.29], 6.0 times more likely to have a baby with low birthweight [95% CI: 2.82–12.76], and 6.1 times more likely to have their newborn admitted to a NICU [95% CI: 2.65–13.83]. Similarly, patients with SMM due to infection were 4.0 [95% CI: 1.42–11.01] and 4.1 [95% CI: 1.44–11.69] times more likely to deliver preterm and have their newborn admitted to NICU, respectively. Having other primary causes of SMM was associated with 2 to 2.2 times higher odds of all three adverse newborn outcomes of interest when compared to patients with SMM from obstetric hemorrhage. Compared to patients whose SMM occurred antepartum, those with intrapartum and postpartum SMM had lower adjusted odds of preterm birth ([AOR = 0.24; 95% CI, 0.09–0.65] and [AOR = 0.26; 95% CI, 0.11–0.60], respectively) and low birthweight ([AOR = 0.37; 95% CI, 0.15–0.93] and [AOR = 0.35; 95% CI, 0.16–0.76], respectively). Similarly, patients with postpartum SMM had lower adjusted odds of NICU admission compared to those with antepartum SMM (AOR = 0.43; 95% CI, 0.19–0.98).

## 4. Discussion

Our analysis finds that the three adverse newborn outcomes of interest were more prevalent among Medicaid than commercially insured patients who experience SMM during the delivery hospitalization. The odds of NICU admission among Medicaid patients remained significantly higher than those with commercial insurance after adjustment for maternal and newborn factors and characteristics of the SMM event, but not for preterm birth and low birthweight. Unlike preterm birth and low birthweight outcomes, NICU admission is influenced by hospital-level factors and practices. Residual differences observed in NICU admission by insurance status may be a reflection of institutional practices among hospitals with a higher proportion of Medicaid patients rather than underlying severity of the neonatal condition.

In adjusted models, covariates associated with odds of these three adverse newborn outcomes included the primary cause of SMM, timing of SMM, and maternal comorbidity index score. Specifically, SMM due to hypertensive disorders was associated with a higher risk of adverse newborn outcomes than obstetric hemorrhage, likely because many hypertensive disorders occur earlier in pregnancy when they have a greater potential to influence neonatal development or result in preterm delivery. This is supported by the finding that antepartum SMM was associated with the highest risk of adverse newborn outcomes, compared to intrapartum and postpartum SMM. The association between higher maternal comorbidity index and adverse newborn outcomes demonstrates that women with more complex underlying health conditions are at higher risk for adverse outcomes, which is well supported by prior research. Taken together, these findings suggest that the observed differences in newborn outcomes are largely driven by maternal health and outcomes and not by insurance type or other socio-demographic factors alone.

Importantly, our models do not include measures of education or income because they are not available in our data, so it is likely that insurance status is serving as a proxy for socioeconomic status. Observed differences in outcomes likely reflect these underlying factors. Taken together, our findings underscore the need for targeted interventions to address both medical and social determinants contributing to maternal morbidity and adverse newborn outcomes.

Prior studies have also shown heightened risk of adverse outcomes among both SMM patients [5,6,7] and patients with Medicaid [10,11,12,13,14,15], separately. Furthermore, a recent study conducted in Illinois showed that deliveries complicated by SMM due to preeclampsia/eclampsia and infection/sepsis were more likely to result in adverse newborn outcomes than those caused by hemorrhage [7]. With obstetric hemorrhage being the most common SMM cause for both Medicaid and commercially insured patients, it is important to recognize antepartum complications like preeclampsia that increase the risk of hemorrhage. Notably, despite lower comorbidity scores and similar cesarean delivery distribution, commercially insurance patients experienced SMM due to obstetric hemorrhage more frequently than patients with Medicaid, being more likely to receive ≥4 units of blood products during hospitalizations with SMM. Maternal multimorbidity is known to significantly increase the risk of adverse birth outcomes as reported by a systematic review of 21 studies from 13 countries across Africa, Asia, Europe, and North America [22]. Similarly, a U.S. National Inpatient Sample analysis documented that women with multiple chronic conditions face heightened risks of preterm delivery, cesarean delivery, and SMM compared to those with one or no chronic conditions [23].

Early screening and managing comorbidities in a timely manner is critical in mitigating adverse outcomes, but late enrollment in Medicaid during the prenatal period poses a challenge. In our study, patients with Medicaid, on average, accessed prenatal care later in comparison to those with commercial insurance, despite Maryland’s Medicaid retroactive eligibility policy—under which patient services provided up to three calendar months prior to the application month are covered through reimbursement to providers [24,25]. This trend has been demonstrated previously—between 2006 and 2023, only 56.3% of patients with births covered by Medicaid in Maryland initiated prenatal care in the first trimester compared to 83.9% of women with commercial insurance [26]. Medicaid enrollment policies may factor into this dynamic. Most women do not qualify for Medicaid until after they confirm a positive pregnancy test and, in addition, the Medicaid application process can take up to 45 days for pregnant women. With later enrollment, they may enter pregnancy with undiagnosed comorbidities that contribute to adverse outcomes for both the mother, the fetus, and newborn. Early prenatal care enables screening and management of comorbidities, potentially reducing the risk of pregnancy and delivery complications.

Overall, Medicaid plays a crucial role in covering low-income populations who have historically faced instability in insurance coverage [27,28]. The benefits of Medicaid expansion are evident in many states. For example, Texas saw a gradual decrease in preterm birth and a reduction in low birthweight for White newborns only [29], while Oregon reported improvement in low-birthweights and preterm births among all newborns irrespective of race, ethnicity, or other characteristics [30]. A recent comparative study between Maryland (an expansion state) and Georgia (a non-expansion state) showed that Maryland had better newborn outcomes, including lower rates of NICU admissions [28]. Beyond perinatal care, Medicaid plays a vital role in addressing broader health challenges. For example, Medicaid covers up to 80.5% of pregnant women with opioid use disorder (OUD) in the US [31], and a study conducted in 47 states in the US on pregnant women with OUD from 2016 to 2018 found that higher SMM rates were associated with lower duration of Medicaid enrollment, highlighting the critical role of Medicaid in this high-risk population [32].

Of note, we also examined ART use among patients with SMM in our data, which is known to be associated with an increased risk of SMM [33,34,35,36] as well as adverse newborn outcomes [37,38,39], particularly among births conceived through multiple embryo transfers. However, we found a lower risk of adverse neonatal outcomes among patients who used ART in our surveillance data. This is likely because of unmeasured characteristics that may have confounded the relationship between ART use and adverse neonatal outcomes, particularly in a sample already restricted to those experiencing SMM. In particular, ART is a marker of access to higher levels of care resources and is likely acting as a proxy for healthcare access rather than an indication that use of ART is protective against adverse neonatal outcomes.

## 5. Strengths and Limitations

Maryland is the only state with a systematic process for identifying and reviewing all SMM events that occur during pregnancy and up to 42 days postpartum in participating birthing hospitals. The data collected and utilized in this study are considered gold standard for assessing SMM and other maternal, fetal, and newborn outcomes in patients with SMM. Neonatal outcomes have not been often explored among SMM patients by insurance status, making this study a valuable contribution to the literature.

Our study has several limitations. Findings may not be generalizable outside the state, as Medicaid coverage varies significantly across the country. However, Maryland’s Medicaid program includes a racially and socioeconomically diverse population, making our findings relevant to similar urban and suburban settings. Participation of hospitals in Maryland’s SMM surveillance is voluntary and was limited to 15 hospitals before state legislation came into effect in October 2024 requiring all hospitals to participate. However, non-participating hospitals were smaller and offered lower levels of maternity care, being overall less likely to have SMM events that met our case definition for surveillance. We did not have information about causes of NICU admission, which could have confounded our analyses. Though data abstraction methods are highly standardized across hospitals, we have not assessed inter-rater reliability on identifying cases of SMM or the covariates included in the analysis. Additionally, due to sample size constraints we assessed each adverse newborn outcome independently though acknowledge that infants who are preterm and low birthweight are conceptually distinct from infants who are full-term and low birthweight.

## 6. Conclusions

Adverse newborn outcomes were more common among Medicaid beneficiaries than commercially insured patients with SMM, with significantly higher adjusted odds of NICU admission. Our findings highlight the need for comprehensive prenatal and postpartum care, improved healthcare access, and targeted interventions to manage maternal health conditions, particularly among Medicaid patients, to improve maternal and newborn health and outcomes. Policies that improve Medicaid access to low-income women are critical. For example, barriers and delays to Medicaid enrollment after the onset of pregnancy can lead to delayed onset of prenatal care, which may prevent providers from being able to identify and manage complications early in pregnancy.

## Figures and Tables

**Table 1:** Maternal, delivery and newborn characteristics among patients with severe maternal morbidity in Maryland: 2020–2023.

		Insurance Type		*p*-Value ^a^
	Total N (%)	Medicaid N (%)	Commercial N (%)	
Maternal Characteristics	N = 588	N = 265	N = 323	
**Age at admission (years)**				<0.001
<25	78 (13.3)	54 (20.4)	24 (7.4)	
25–34	276 (46.9)	134 (50.6)	142 (44.0)	
35+	234 (39.8)	77 (29.1)	157 (48.6)	
**Race and ethnicity**				<0.001
Black, Non-Hispanic	274 (46.6)	160 (60.4)	114 (35.3)	
White, Non-Hispanic	202 (34.4)	53 (20.0)	149 (46.1)	
Hispanic	56 (9.5)	39 (14.7)	17 (5.3)	
Asian	33 (5.6)	4 (1.5)	29 (9.0)	
Multi-race/Other/Unknown	23 (3.9)	9 (3.4)	14 (4.3)	
**Parity, median (IQR)**	1 (0–2)	2 (0–3)	1 (0–2)	<0.001
**Timing of prenatal care initiation**				<0.001
1st trimester	384 (65.3)	139 (52.5)	245 (75.9)	
2nd or 3rd trimester	100 (17.0)	63 (23.8)	37 (11.5)	
No prenatal care	28 (4.8)	20 (7.5)	8 (2.5)	
Timing of prenatal care unknown	76 (12.9)	43 (16.2)	33 (10.2)	
**Comorbidity index score, median (IQR)**	12.0 (4.0–21.5)	14.5 (8.0–22.5)	9.0 (3.0–19.5)	<0.001
**ART use during index pregnancy ^b^**	66 (11.2)	9 (3.4)	57 (17.6)	<0.001
**Delivery and newborn/fetal characteristics**	**n = 467**	**n = 213**	**n = 254**	
**Mode of delivery**				0.94
Cesarean delivery	343 (73.4)	157 (73.7)	186 (73.2)	
Spontaneous vaginal delivery	99 (21.2)	46 (21.6)	53 (20.9)	
Assisted vaginal delivery	7 (1.5)	3 (1.4)	4 (1.6)	
Surgical evacuation ^c^	18 (3.9)	7 (3.3)	11 (4.3)	
**Delivery outcome**				0.21
Live birth	421 (90.1)	188 (88.3)	233 (91.7)	
Fetal death	46 (9.9)	25 (11.7)	21 (8.3)	
**Congenital anomaly**	14 (3.0)	6 (2.8)	8 (3.1)	0.83
**Newborn/fetal sex ^d^**				0.42
Female	234 (50.1)	102 (47.9)	132 (52.0)	
Male	209 (44.8)	103 (48.4)	106 (41.7)	
**Gestational age at birth median (IQR) ^e^**	37 w0 d (33 w2 d–39 w0 d)	35 w5 d (31 w5 d–38 w4 d)	37 w4 d (34 w4 d–39 w2 d)	0.002
**Birthweight (g), median (IQR) ^f^**	2880.0 (1990.0–3430.0)	2490.0 (1720.0–3235.0)	3116.5 (2325.0–3570.0)	<0.001
**Newborn outcomes ^g^**	**n = 421**	**n = 188**	**n = 233**	
**Preterm birth (<37 weeks gestational age) ^h^**	187 (44.7)	107 (56.9)	80 (34.8)	<0.001
**Low birthweight (<2500 g) ^i^**	156 (37.6)	89 (47.6)	67 (29.4)	<0.001
**NICU admission ^j^**	210 (50.7)	117 (63.6)	93 (40.4)	<0.001

Notes: IQR = Interquartile ranges, ART = assisted reproductive technology, w = weeks, d = days, g = grams, NICU = Neonatal Intensive Care Unit. ^a^ *p*-values assess differences between patients with Medicaid and private insurance using χ^2^ analyses for categorical variables and *t*-tests for continuous variables. ^b^ Use of ART unknown for 56 patients (9.5%). ^c^ Surgical evacuation (of the uterus) includes dilation and curettage (D&C), salpingectomy, and surgery for ectopic pregnancy or ruptured uterus. ^d^ Neonatal sex was undifferentiated among 13 (2.8%) newborn/fetuses and missing among 11 (2.4%). ^e^ Gestational age was missing for two live births (0.48%) and three fetal deaths. ^f^ Birthweight was missing among six live births (1.43%) and 12 fetal deaths. ^g^ Status combined in case of twin or higher order pregnancies. ^h^ Preterm status was missing among two live births (0.48). ^i^ Low birthweight was missing among six live births (1.43%). ^j^ NICU admission was missing among seven live births (1.66%).

**Table 2:** Characteristics of hospitalizations with severe maternal morbidity by insurance type in Maryland: 2020–2023.

Characteristics		Insurance Type		*p*-Value ^a^
	Total N (%)	Medicaid N (%)	Commercial N (%)	
	N = 588	N = 265	N = 323	
**SMM occurred during delivery hospitalization**	467 (79.4)	213 (80.4)	254 (78.6)	0.60
**Length of stay (days)**				
Mean (SD)	6.9 (7.4)	7.9 (9.4)	6.1 (5.2)	0.002
Median (IQR)	5 (4–8)	5 (4–8)	5 (3–7)	0.002
**ICU/CCU admission**	376 (63.9)	179 (67.5)	197 (61.0)	0.099
**≥4 units blood products transfused**	357 (60.7)	140 (52.8)	217 (67.2)	<0.001
**Timing of SMM**				0.040
Antepartum	146 (24.8)	79 (29.8)	67 (20.7)	
Intrapartum	79 (13.4)	33 (12.5)	46 (14.2)	
Postpartum	363 (61.7)	153 (57.7)	210 (65.0)	
**Primary cause of SMM**				0.001
Obstetric hemorrhage	329 (56.0)	125 (47.2)	204 (63.2)	
Infection/sepsis ^b^	64 (10.9)	32 (12.1)	32 (9.9)	
Hypertensive disorders of pregnancy	63 (10.7)	29 (10.9)	34 (10.5)	
Cardiovascular condition	30 (5.1)	20 (7.5)	10 (3.1)	
Hematologic condition ^c^	18 (3.1)	10 (3.8)	8 (2.5)	
Other condition ^d^	84 (14.3)	49 (18.5)	35 (10.8)	
**SMM was preventable ^e^**	199 (33.8)	88 (33.2)	111 (34.4)	0.77
With changes in provider-level factors	139 (23.6)	53 (20.0)	86 (26.6)	0.060
With changes in system-level factors	84 (14.3)	42 (15.8)	42 (13.0)	0.33
With changes in patient-level factors	90 (15.3)	52 (19.6)	38 (11.8)	0.008

Notes: SMM = severe maternal morbidity, SD = standard deviation, IQR = interquartile range, ICU/CCU = intensive care unit/critical care unit. ^a^ *p*-values assess differences between patients with Medicaid and commercial insurance using χ^2^ analyses for categorical variables and *t*-test for continuous variables. ^b^ Infections include COVID-19. ^c^ Hematologic conditions include sickle cell disease, hemophilia, thrombocytopenia, anemia, and thrombotic thrombocytopenic purpura. ^d^ Other conditions include adverse drug reaction, anesthesia complications, apnea, cancer, embolism, gastrointestinal disorders, injury, metabolic/endocrine conditions, neurologic conditions, pulmonary conditions, renal disease, and musculoskeletal conditions. ^e^ SMM events were considered preventable if there were one or more condition(s)/factor(s) during the antepartum, intrapartum, and/or postpartum periods that, if changed, could have altered the SMM outcome.

**Table 3:** Odds of adverse delivery and newborn outcomes among deliveries complicated by severe maternal morbidity in Maryland: 2020–2023.

Characteristics	Preterm Birth (<37 w) ^ab^				Low Birthweight (<2500 gm) ^cd^				NICU Admission ^ef^			
	Unadjusted		Adjusted		Unadjusted		Adjusted		Unadjusted		Adjusted	
	OR	95% CI	OR	95% CI	OR	95% CI	OR	95% CI	OR	95% CI	OR	95% CI
**Medicaid** (Commercial insurance = ref)	**2.45**	**1.65–3.63**	1.62	0.94–2.80	**2.18**	**1.46–3.27**	1.33	0.78–2.28	**2.57**	**1.73–3.84**	**1.83**	**1.09–3.07**
**Race and ethnicity** (White non-Hispanic = ref)												
Black, Non-Hispanic	**1.76**	**1.14–2.73**	0.84	0.47–1.49	**1.72**	**1.10–2.71**	0.93	0.53–1.64	1.50	0.97–2.31	0.73	0.42–1.26
Hispanic	1.15	0.58–2.26	1.16	0.49–2.72	1.60	0.8–3.18	1.78	0.78–4.1	1.14	0.58–2.27	0.95	0.42–2.16
Asian	**0.38**	**0.15–0.98**	0.52	0.17–1.59	0.42	0.15–1.17	0.68	0.22–2.13	0.49	0.21–1.14	0.74	0.28–1.92
Multi-race/Other/Unknown	1.08	0.33–3.55	1.11	0.26–4.76	0.70	0.18–2.71	0.60	0.12–2.94	2.29	0.66–7.92	2.40	0.62–9.27
**Parity**	**1.21**	**1.08–1.37**	1.07	0.92–1.24	1.10	0.99–1.23	0.96	0.83–1.1	**1.19**	**1.06–1.34**	1.09	0.95–1.26
**Comorbidity Index**	**1.08**	**1.06–1.10**	**1.09**	**1.07–1.11**	**1.06**	**1.04–1.08**	**1.08**	**1.05–1.10**	**1.05**	**1.03–1.07**	**1.05**	**1.03–1.07**
**Use of ART** (No = ref)	0.73	0.41–1.3	0.93	0.44–1.95	**0.47**	**0.24–0.91**	0.49	0.22–1.07	**0.56**	**0.31–0.99**	0.73	0.37–1.44
**Newborn female sex** (Male = ref)	1.02	0.69–1.50	1.21	0.74–1.97	1.03	0.69–1.54	1.23	0.76–2.00	0.74	0.50–1.10	0.77	0.49–1.22
**Primary cause of SMM** (Obstetric hemorrhage = ref)												
Hypertensive disorders of pregnancy	**4.71**	**2.41–9.18**	**4.75**	**2.19–10.29**	**6.00**	**3.09–11.66**	**6.00**	**2.82–12.76**	**6.52**	**3.03–14.01**	**6.06**	**2.65–13.83**
Infections ^g^	**3.77**	**1.63–8.71**	**3.95**	**1.42–11.01**	**2.45**	**1.07–5.60**	2.69	0.97–7.40	**4.30**	**1.76–10.52**	**4.10**	**1.44–11.69**
Other ^h^	**2.49**	**1.47–4.23**	**2.06**	**1.03–4.13**	**2.72**	**1.59–4.66**	**2.21**	**1.12–4.36**	**2.45**	**1.43–4.20**	**1.96**	**1.03–3.72**
**Timing of SMM** (Antepartum = ref)												
Intrapartum	**0.24**	**0.11–0.53**	**0.24**	**0.09–0.65**	**0.34**	**0.16–0.71**	**0.37**	**0.15–0.93**	**0.42**	**0.19–0.92**	0.69	0.27–1.78
Postpartum	**0.17**	**0.09–0.33**	**0.26**	**0.11–0.60**	**0.23**	**0.12–0.42**	**0.35**	**0.16–0.76**	**0.21**	**0.11–0.41**	**0.43**	**0.19–0.98**

Notes: SMM = severe maternal morbidity, ART = assisted reproductive technology, NICU = neonatal intensive care unit, g = grams, OR = odds ratio, CI = confidence interval; *p* < 0.05 are shown in bold. ^a^ Preterm birth status missing among two newborns. ^b^ Model includes indicator for unknown/undifferentiated sex for 6 (1.4%) newborns, of which three born preterm in addition to all other factors shown. ^c^ Low birthweight status missing for six newborns; ^d^ Model includes indicator for unknown/undifferentiated sex for four (1.0%) newborns, of which one was low birthweight in addition to all other factors shown. ^e^ NICU status missing for seven newborns. ^f^ Model includes indicator for unknown/undifferentiated sex of four (1.0%) newborns, of which three were admitted to an NICU in addition to all other factors shown. ^g^ Infections include COVID-19 infections. ^h^ Other conditions include adverse drug reaction, anesthesia complications, apnea, cancer, embolism, gastrointestinal disorders, injury, metabolic/endocrine conditions, neurologic conditions, pulmonary conditions, renal disease, and musculoskeletal conditions.

## Data Availability

The datasets presented in this article are not readily available because they include confidential and protected information.

## Supplementary Materials

The following supporting information can be downloaded at: https://www.mdpi.com/article/10.3390/healthcare14060804/s1, Table S1: Maternal comorbidity index and distribution of conditions by insurance type; Table S2: Distribution of adverse delivery and newborn outcomes among deliveries complicated by severe maternal morbidity in Maryland: 2020–2023.
