# Supplementary material for: Adverse Newborn Outcomes by Insurance Status Among Patients with Severe Maternal Morbidity in Maryland: 2020–2023
Source: Healthcare (Basel). 2026 Mar 21;14(6):804. doi: 10.3390/healthcare14060804 (PMC13027368; doi:10.3390/healthcare14060804)
Supplement: Supplementary file 1 [file healthcare-14-00804-s001.zip › healthcare-4032720-supplementary.pdf]

SUPPLEMENTAL TABLE S1: Maternal comorbidity index and distribution of conditions by insurance type

					Insurance type		
Conditions from Leonard et al. (2022)	Index weight from Leonard et al. (2022)	Variations for present study	Present study scoring	Total N (%) N=588	Medicaid N (%) N=265	Commercial N (%) N=323	p-value [a]
Anemia, preexisting	9		9	82 (13.9)	47 (17.7)	35 (10.8)	0.016
Asthma, acute or moderate/severe	5		5	107 (18.2)	61 (23.0)	46 (14.2)	0.006
Bleeding disorder, preexisting	9		9	9 (1.5)	3 (1.1)	6 (1.9)	0.48
Cardiac disease, preexisting	14		14	41 (7.0)	21 (7.9)	20 (6.2)	0.41
Chronic hypertension	3		3	110 (18.7)	57 (21.5)	53 (16.4)	0.11
Chronic renal disease	17		17	10 (1.7)	5 (1.9)	5 (1.5)	0.75
Connective tissue or autoimmune disease	4		4	13 (2.2)	4 (1.5)	9 (2.8)	0.29
Delivery BMI ≥ 40	1	Pre-pregnancy BMI ≥ 30	1	216 (36.7)	108 (40.8)	108 (33.4)	0.067
Gastrointestinal disease	3		3	16 (2.7)	7 (2.6)	9 (2.8)	0.91
Gestational diabetes mellitus	1		1	68 (11.6)	27 (10.2)	41 (12.7)	0.34
HIV/AIDS	13		13	4 (0.7)	1 (0.4)	3 (0.9)	0.42
Major mental health disorder	3		3	202 (34.4)	102 (38.5)	100 (31.0)	0.056
Neuromuscular disease	6		6	14 (2.4)	8 (3.0)	6 (1.9)	0.36
Placenta accreta spectrum	27		27	52 (8.8)	19 (7.2)	33 (10.2)	0.20
Placenta previa, complete or partial	8		8	30 (5.1)	8 (3.0)	22 (6.8)	0.038
Placental abruption	9		9	46 (7.8)	29 (10.9)	17 (5.3)	0.011
Preeclampsia with severe features	12	Preeclampsia with and without severe features	8.5 (averaged)	156 (26.5)	83 (31.3)	73 (22.6)	0.017
Preeclampsia without severe features or gestational hypertension	5	Gestational hypertension	5	22 (3.7)	9 (3.4)	13 (4.0)	0.69

Preexisting diabetes mellitus	2		2	38 (6.5)	21 (7.9)	17 (5.3)	0.19
Preterm birth (<37 weeks)	8	Prior Preterm Birth	8	79 (13.4)	55 (20.8)	24 (7.4)	<0.001
Previous cesarean birth	2		2	169 (28.7)	90 (34.0)	79 (24.5)	0.011
Pulmonary hypertension	20		20	7 (1.2)	3 (1.1)	4 (1.2)	0.91
Substance use disorder	4		4	74 (12.6)	65 (24.5)	9 (2.8)	<0.001
Thyrotoxicosis	2		2	8 (1.4)	6 (2.3)	2 (0.6)	0.087
Twin/multiple pregnancy	9		9	30 (5.1)	10 (3.8)	20 (6.2)	0.18
Uterine fibroids	4		4	40 (6.8)	12 (4.5)	28 (8.7)	0.047
Maternal age ≥ 35 years	0		0	234 (39.8)	77 (29.1)	157 (48.6)	<0.001
**Total score (median, IQR)**	**200**	**-**	**188**	12.00 (4.00-21.50)	14.50 (8.00-22.50)	9.00 (3.00-19.50)	<0.001

Notes:
BMI=Body mass index, HIV/AIDS=Human Immunodeficiency Virus Infection/Acquired Immune Deficiency Syndrome; SD=Standard deviation
[a]P-value assesses differences between patients with Medicaid and private insurance using χ^2 analyses.

SUPPLEMENTAL TABLE S2: Distribution of adverse delivery and newborn outcomes among deliveries complicated by severe maternal morbidity in Maryland: 2020-2023.

Characteristics	Preterm birth (<37 weeks)[a]		Low birthweight (<2,500g)[b]		NICU admission[c]	
	No N(%)	Yes N(%)	No N(%)	Yes N(%)	No N(%)	Yes N(%)
	N=231	N=188	N=259	N=156	N=204	N=210
**Insurance type**						
Commercial	150 (64.9)	81 (43.1)	161 (62.2)	67 (42.9)	137 (67.2)	93 (44.3)
Medicaid	81 (35.1)	107 (56.9)	98 (37.8)	89 (57.1)	67 (32.8)	117 (55.7)
**Race and ethnicity**						
White, Non-Hispanic	92 (39.8)	61 (32.4)	101 (39.0)	48 (30.8)	80 (39.2)	70 (33.3)
Black, Non-Hispanic	83 (35.9)	97 (51.6)	99 (38.2)	81 (51.9)	78 (38.2)	102 (48.6)
Hispanic	25 (10.8)	19 (10.1)	25 (9.7)	19 (12.2)	21 (10.3)	21 (10.0)
Asian	24 (10.4)	6 (3.2)	25 (9.7)	5 (3.2)	21 (10.3)	9 (4.3)
Multi-race/Other/Unknown	7 (3.0)	5 (2.7)	9 (3.5)	3 (1.9)	4 (2.0)	8 (3.8)
**Parity**	1 (0-2)	1 (0-3)	1 (0-2)	1 (0-3)	1 (0-2)	1 (0-3)
**Comorbidity index score, median (IQR)**	8.5 (3.0-14.5)	21.0 (12.0-32.5)	9.0 (3.0-16.5)	21.0 (12.0-32.0)	9.0 (3.0-16.0)	18.0 (9.0-29.5)
**Use of ART**						
No	197 (85.3)	167 (88.8)	217 (83.8)	143 (91.7)	170 (83.3)	189 (90.0)
Yes	34 (14.7)	21 (11.2)	42 (16.2)	13 (8.3)	34 (16.7)	21 (10.0)
**Newborn sex**						
Male	107 (46.3)	86 (45.7)	121 (46.7)	72 (46.2)	88 (43.1)	105 (50.0)
Female	121 (52.4)	99 (52.7)	135 (52.1)	83 (53.2)	115 (56.4)	102 (48.6)
Unknown sex	3 (1.3)	3 (1.6)	3 (1.2)	1 (0.6)	1 (0.5)	3 (1.4)
**Primary cause of SMM**						
Obstetric hemorrhage	177 (76.6)	94 (50.0)	196 (75.7)	74 (47.4)	161 (78.9)	107 (51.0)

Hypertensive disorders of pregnancy	14 (6.1)	35 (18.6)	15 (5.8)	34 (21.8)	9 (4.4)	39 (18.6)
Infections	9 (3.9)	18 (9.6)	13 (5.0)	12 (7.7)	7 (3.4)	20 (9.5)
Other[d]	31 (13.4)	41 (21.8)	35 (13.5)	36 (23.1)	27 (13.2)	44 (21.0)
**Timing of SMM**						
Antepartum	13 (5.6)	46 (24.5)	19 (7.3)	38 (24.4)	13 (6.4)	46 (21.9)
Intrapartum	36 (15.6)	31 (16.5)	40 (15.4)	27 (17.3)	27 (13.2)	40 (19.0)
Postpartum	182 (78.8)	111 (59.0)	200 (77.2)	91 (58.3)	164 (80.4)	124 (59.0)

Notes:
SMM=Severe Maternal Morbidity, NICU=Neonatal Intensive Care Unit, ART=Assisted Reproductive Technology, g=grams, IQR=interquartile range, CI=Confidence Interval; P<0.05 are shown in bold.
[a]Preterm birth status missing among 2 newborns; [b]Low birthweight status missing for 6 newborns; [c]NICU status missing for 7 newborns; [d]Other conditions include adverse drug reaction, anesthesia complications, apnea, cancer, embolism, gastrointestinal disorders, injury, metabolic/endocrine conditions, neurologic conditions, pulmonary conditions, renal disease, musculoskeletal conditions.
